# Editorial: Shaping cities for health: built environment, active living, and well-being

**DOI:** 10.3389/fspor.2026.1914249

**Published:** 2026-07-13

**Authors:** Kaichen Zhou, Izzy Yi Jian, Xiji Jiang

**Affiliations:** 1 College of Architecture and Urban Planning, Tongji University, Shanghai, China; 2 Department of Social Sciences and Policy Studies, The Education University of Hong Kong, Hong Kong SAR, China; 3 College of Architecture, Xi'an University of Architecture and Technology, Xi'an, China

**Keywords:** active living, built environment, environmental exposure, health equity, healthy cities

## Introduction

Urbanization, aging, environmental exposure, and shifting lifestyls have brought the relationship between environment and health into sharp focus. Sedentary behavior, physical inactivity, mental health challenges, and unequal access to health-promoting spaces are not merely matters of individual choice—they are embedded in where people live. The built environment, through parks, streets, transport, schools, and public spaces, shapes whether physical activity can be safe, convenient, and consistent. The question, then, is no longer whether cities influence health, but how urban environments can be organized to support more equitable and sustainable well-being.

This special issue approaches that question through a socio-ecological lens, in which health and well-being are understood as emerging from the interaction between individuals and nested social and physical systems in which they are situated (1–3). The four papers trace a connected line of inquiry: from environmental exposure, to built-social environment interaction, to community implementation and governance, and finally to health equity. Reading together, they make a cumulative argument that healthy cities depend on coordinated actions across environmental, social, community, and governance dimensions, and that interventions targeting any single level in isolation are likely to fall short.

The Research Topic includes four contributions spanning the socio-ecological model. Hanami et al. investigate indoor exposure to microplastics among students in Makassar, Indonesia. Zhang et al. examine how built and social environments jointly shape older adults' mental health in Guangzhou, China. Burke et al. report on a participatory initiative to expand opportunities for movement in rural aging communities in Nova Scotia, Canada. de Farias et al. analyze long-term trends in health perceptions among Brazilian adults from 2006 to 2023. Together, these studies trace how healthy environments are experienced as exposures, socially mediated, implemented through community action, and reflected in patterns of health inequality.

Hanami et al. analyze the level of environmental exposure by examining indoor microplastic fallout in school classrooms in Makassar, Indonesia. Through passive sampling in six schools on both weekdays and weekends, the study characterizes the abundance, shape, color, size, and potential inhalation risk of falling microplastics. The findings show that indoor microplastic concentrations were generally higher on school days, with the highest concentration reaching 10,786 MPs/m^2^/day. Fragments and fibers were the dominant forms, most particles were smaller than 500 *μ*m, and estimated annual exposure among students ranged from 0.025 to 0.115 mg/kg-Bw/year. By focusing on classrooms, Hanami et al. extend the discussion of healthy built environments beyond outdoor walkability, parks, and transport systems to include indoor spaces where children and adolescents spend substantial amounts of time. Their study highlights that shaping cities for health requires not only creating opportunities for movement and activity but also identifying and reducing environmental risks embedded in everyday settings.

Zhang et al. move the discussion to the interaction between built and social environments by examining the mental health of older adults in Guangzhou, China. Drawing on Baidu point-of-interest data and questionnaire survey data from 20 communities, the study applies multilevel mediation models to investigate how community environments are associated with older adults’ mental health. The results show that built-environment factors, including park accessibility, distance to the nearest park, and distance to the nearest public transit station, are significantly associated with mental health outcomes. More importantly, the study demonstrates that social capital and community safety mediate these relationships and that the pathways differ across income groups. In doing so, Zhang et al. show that health-promoting and age-friendly communities cannot rely solely on physical “hardware” such as parks, transit access, and spatial proximity; they also require social “software” such as trust, safety, social capital, and community connection.

Burke et al. translate these environmental concerns into questions of community-based implementation and governance. Their community case study documents *Communities on the Move*, a four-year initiative in Nova Scotia, Canada, designed to increase low-barrier and less-structured opportunities for daily movement in rural and aging communities. The initiative integrates policy support, built-environment improvements, social-environment actions, public engagement, and cross-sector partnerships. Phase 1 findings show that most participating communities formed leadership teams, developed action plans, delivered social programs, improved infrastructure, and launched engagement activities such as *Make Your Move*. Although one community experienced delays in leadership formation, existing local committees and partners helped sustain implementation. This case study demonstrates that promoting active living involves more than building sidewalks, trails, or cycling facilities; it also depends on local leadership, community participation, flexible partnerships, and the governance capacity to translate healthy-city principles into practice.

de Farias et al. extend the narrative to health equity and population-level well-being by analyzing long-term variation in health perception among Brazilian adults from 2006 to 2023. Using data from 799,695 participants and multilevel modeling, the study examines how self-rated health varies by year, region, BMI, education, sex, and exercise duration. The findings indicate that health perception declined over time and differed substantially across Brazilian regions, BMI categories, education levels, and physical activity patterns. Individuals with a healthy weight, higher education, and more than 30 min of exercise reported better health perception, whereas no substantial difference emerged between women and men. These results highlight the persistence of structural inequalities in health and reinforce the need for public policies that expand access to safe, high-quality, and preferably free public spaces for physical activity. In this way, de Farias et al. link individual health perception to the broader social and spatial conditions that shape active living, well-being, and health equity.

Taken together, these contributions demonstrate that shaping cities for health should be understood not as a narrow matter of spatial planning, but as a multidimensional public health and governance agenda. From a socio-ecological perspective, active living and well-being are shaped by the environments to which people are routinely exposed, the social processes through which built environments are experienced and mobilized, the community capacities that enable evidence-informed interventions, and the policy arrangements that govern the distribution of health-promoting resources and environmental risks. Collectively, these contributions highlight the importance of moving beyond isolated physical interventions toward more integrated approaches that connect environmental exposure, interactions between the built and social environments, community governance, and health equity. Advancing such integrated approaches will be essential for supporting healthy behaviors, protecting vulnerable populations, narrowing spatial and social inequalities, and building more inclusive and sustainable forms of urban well-being.
